# Author Correction: Toxin-mediated depletion of NAD and NADP drives persister formation in a human pathogen

**DOI:** 10.1038/s44318-024-00354-4

**Published:** 2025-04-22

**Authors:** Isabella Santi, Raphael Dias Teixeira, Pablo Manfredi, Hector Hernandez Gonzalez, Daniel C Spiess, Guillaume Mas, Alexander Klotz, Andreas Kaczmarczyk, Simon van Vliet, Nicola Zamboni, Sebastian Hiller, Urs Jenal

**Affiliations:** 1 Biozentrum, University of Basel, Basel, Switzerland; 2 Institute of Molecular Systems Biology, ETH Zürich, Zürich, Switzerland; 3 Present Address: Department for Biosystems Science and Engineering, ETH Zürich, Basel, Switzerland

## Abstract

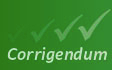

**Correction to:** *The EMBO Journal* (2024) 43:5211–5236. 10.1038/s44318-024-00248-5 | Published online 25 September 2024

**The author list is corrected**.

Dr. Simon van Vliet is added to the paper as the 9th author, with the affiliation: Biozentrum, University of Basel, Basel, Switzerland

All authors agree to this correction.

