# Supplementary material for: Toxin-mediated depletion of NAD and NADP drives persister formation in a human pathogen
Source: EMBO J. 2024 Sep 25;43(21):19. doi: 10.1038/s44318-024-00248-5 (PMC11535050; doi:10.1038/s44318-024-00248-5)
Supplement: Supplementary file 1 — Appendix [file 44318_2024_248_MOESM1_ESM.pdf]

Toxin-mediated depletion of NAD and NADP drives persister formation in a human pathogen

Isabella Santi[1*], Raphael Dias Teixeira[1*], Pablo Manfredi[1], Hector Hernandez Gonzalez[1], Daniel C. Spiess[1], Guillaume Mas[1], Alexander Klotz[1,2], Andreas Kaczmarczyk[1], Nicola Zamboni[3], Sebastian Hiller[1] and Urs Jenal[1]

* Isabella Santi and Raphael Dias Teixeira contributed equally to this work.

Appendix

Table of contents

Appendix Table S1. Strains used in this study **2**
Appendix Table S2. Plasmids used in this study **4**
Appendix Table S3. Oligos used in this study **7**
Appendix Table S4. Crystallographic data collection and refinement statistics **9**
Appendix Figure S1. Phylogenetic tree of bacterial RES domain proteins **11**
References **12**

Appendix Table S1. Strains used in this study

Strains	Description	Reference
PAO1	*P. aeruginosa* wild type	1
$natT_{E29D}$	Chromosomal point mutation of the *natT* gene at the original *natT* locus	2
$natT_{E29D\ R82A}$	Chromosomal point mutation of the *natT* gene at the original *natT* locus	This study
Δ*natT*	Unmarked deletion of *natT* (PA1030) in PAO1	This study
Δ*natR*	Unmarked deletion of *natR* (PA1029) in PAO1	This study
Δ*natRT*	Unmarked deletion of *natR* (PA1029) and *natT* (PA1030) in PAO1	This study
Δ*natR* $natT_{E29D}$	Unmarked deletion of *natR* (PA1029) in $natT_{E29D}$ background	This study
$natRT_{wt}$*::gfp*	Chromosomal fluorescent transcriptional reporter of *natRT* operon in PAO1	This study
Δ*natR* $natRT_{wt}$*::gfp*	Unmarked deletion of *natR* (PA1029) in $natRT_{wt}$*::gfp* background	This study
Δ*natT* $natRT_{wt}$*::gfp*	Unmarked deletion of *natT* (PA1030) in $natRT_{wt}$*::gfp* background	This study
$natRT_{E29D}$*::gfp*	Chromosomal fluorescent transcriptional reporter of *natRT* operon in $natT_{E29D}$ background	This study
Δ*natR* $natT_{E29D}$*::gfp*	Unmarked deletion of *natR* (PA1029) in $natRT_{E29D}$*::gfp* background	This study
$natT_{wt}$*-FLAG*	PAO1 with $natT_{wt}$*-FLAG*, with the flag tag fused on the 3' end at the original *natT* locus	This study
Δ*natR* $natT_{wt}$*-FLAG*	Unmarked deletion of *natR* (PA1029) in $natT_{wt}$*-FLAG* background	This study
$natT_{E29D}$*-FLAG*	$natT_{E29D}$ with $natT_{E29D}$*-FLAG*, with the flag tag fused on the 3' end at the original *natT* locus.	This study
Δ*natR* $natT_{E29D}$*-FLAG*	Unmarked deletion of *natR* (PA1029) in $natT_{E29D}$*-FLAG* background	This study
pcnB1::gfp	Chromosomal fluorescent transcriptional reporter of *pcnB1* (PA4919) in PAO1	This study
$natT_{E29D}$ *pcnB1::gfp*	Chromosomal fluorescent transcriptional reporter of *pcnB1* (PA4919) in $natT_{E29D}$	This study
Δ*natR* $natT_{E29D\ R82A}$ *pcnB1::gfp*	Unmarked deletion of *natR* (PA1029) in $natT_{E29D}$ *pcnB1::gfp* background	This study
$natT_{E29D\ R82A}$ *pcnB1::gfp*	Chromosomal fluorescent transcriptional reporter of *pcnB1* (PA4919) in $natT_{E29D\ R82A}$ background	This study
ΔrelA spoT pcnB1::gfp	Chromosomal fluorescent transcriptional reporter of *pcnB1* (PA4919) in *ΔrelA spoT* background	This study
$natT_{E29D}$ *ΔrelA spoT pcnB1::gfp*	Chromosomal fluorescent transcriptional reporter of *pcnB1* (PA4919) in $natT_{E29D}$ *ΔrelA spoT* background	This study
pcnB1::gfp ΔnrtR	Unmarked deletion of *nrtR* (PA4916) in *pcnB1::gfp* background	This study
$natT_{E29D}$ *pcnB1::gfp ΔnrtR*	Unmarked deletion of *nrtR* (PA4916) in $natT_{E29D}$ *pcnB1::gfp* background	This study
$natT_{E29D}$ *ΔnrtR*	Unmarked deletion of *nrtR* (PA4916) in $natT_{E29D}$ background	This study

natT$_{E29D}$ *ΔnadD2*	Unmarked deletion of *nadD2* (PA4917) in *natT*$_{E29D}$ background	This study
ΔnadD2	Unmarked deletion of *nadD2* (PA4917) in PAO1 background	This study
ΔrelA spoT	Unmarked deletion of *relA* (PA0934) and *spoT* (PA5338) in PAO1 background	This study
natT$_{E29D}$ *ΔrelA spoT*	Unmarked deletion of *relA* (PA0934) and *spoT* (PA5338) in *natT*$_{E29D}$ background	This study
natT$_{E29D}$*-FLAG ΔrelA spoT*	Unmarked deletion of *relA* (PA0934) and *spoT* (PA5338) in *natT*$_{E29D}$*-FLAG* background	This study
natT$_{E29D}$*::gfp ΔrelA spoT*	Unmarked deletion of *relA* (PA0934) and *spoT* (PA5338) in *natT*$_{E29D}$*::gfp* background	This study
*attb::*TIMER^{bac}	Chromosomal copy of of TIMER^{bac} at *attb* locus	This study
natT$_{E29D}$ *attb::*TIMER^{bac}	*natT*$_{E29D}$ with a chromosomal copy of TIMER^{bac} at *attb* locus	This study
Δpel psl	Unmarked deletion of *pel*, *psl*	3
Δpel psl pilA fliC natT$_{E29D}$ *attb::*TIMER^{bac}	Unmarked deletion of *pel*, *psl*, *pilA*, *fliC* in *natT*$_{E29D}$ background with a chromosomal copy of TIMER^{bac} at *attb* locus	This study
Tn::nadD	*Tn::PA1004*	4
Tn::nadB	*Tn::PA0761*	4
Tn::nadC	*Tn::PA4524*	4
Tn::pcnA	*Tn::PA4918*	4
Tn::pcnB1	*Tn::PA4919*	4
Tn::nadD2	*Tn::PA4917*	4
Tn::nadE	*Tn::PA4920*	4
Tn::pcnB2	*Tn::PA4376 (homologue to PA4919)*	4
Tn::nadD1	*Tn::PA4006 (homologue to PA4917)*	4

Appendix Table S2. Plasmids used in this study

Constructs	Description	Construction	Reference
miniCTX::*pX2-gfp*	mini-CTX for *gfp* integration into *attB* locus under control of the pX2 constitutive promoter (Tet^R)	-	Dirk Bumann
miniCTX::*pX2-mCherry*	mini-CTX for *mCherry* integration into *attB* locus under control of the pX2 constitutive promoter (Tet^R)	-	Dirk Bumann
miniCTX::*pX2-TIMER*^{bac}	mini-CTX for *TIMER*^{bac} integration into *attB* locus under control of the pX2 constitutive promoter (Tet^R)	-	Dirk Bumann
pME6032	Tet^R, P_K, 9.8 kb pVS1 derived shuttle vector	-	5
pME6032::$natT_{wt}$	pME6032 with $natT_{wt}$ under the control of IPTG promoter	*natT* (PA1030) was amplified from PAO1 genomic DNA with primers A and B as *Eco*RI-*Kpn*I fragment	This study
pME6032::$natT_{wt\text{-}ATG}$	pME6032 with $natT_{wt\text{-}ATG}$ under the control of *lac* promoter	*natT* (PA1030) was amplified from PAO1 genomic DNA with primers C and B as *Eco*RI-*Kpn*I fragment	This study
pME6032::$natT_{E29D}$	pME6032 with $natT_{E29D}$ under the control of *lac* promoter	*natT* (PA1030) was amplified from $natT_{E29D}$ genomic DNA with primers A and B as *Eco*RI-*Kpn*I fragment	This study
pME6032::$natT_{E29D\ R82A}$	pME6032 with $natT_{E29D\ R82A}$ under the control of *lac* promoter	*natT* (PA1030) was amplified from $natT_{E29D\ R82A}$ genomic DNA with primers A and B as *Eco*RI-*Kpn*I fragment	This study
pME6032::*natR*	pME6032 with *natR* under the control of *lac* promoter	*natR* (PA1029) was amplified from PAO1 genomic DNA with primers D and E as *Eco*RI-*Kpn*I fragment	This study
pME6032::$natT_{V117A}$	pME6032 with $natT_{V117A}$ under the control of *lac* promoter	*natT* (PA1030) was amplified from CI_1125 genomic DNA with primers A and B as *Eco*RI-*Kpn*I fragment	This study
pME6032::$natT_{I125T}$	pME6032 with $\mathrm{natT}_{\mathrm{I125T}}$ under the control of *lac* promoter	*natT* (PA1030) was amplified from CI_1186 genomic DNA with primers A and B as *Eco*RI-*Kpn*I fragment	This study
pME6032::$\mathrm{natT}_{\mathrm{S170T}}$	pME6032 with $natT_{S170T}$ under the control of *lac* promoter	*natT* (PA1030) was amplified from CI_1129 genomic DNA with primers A and B as *Eco*RI-*Kpn*I fragment	This study
pME6032::$natT_{I14V\ V117A}$	pME6032 with $natT_{I14V\ V117A}$ under the control of *lac* promoter	*natT* (PA1030) was amplified from CI_1107 genomic DNA with primers A and B as *Eco*RI-*Kpn*I fragment	This study
pME6032::$natT_{V117A\ D151G}$	pME6032 with $natT_{V117A\ D151G}$ under the control of *lac* promoter	*natT* (PA1030) was amplified from CI_1115 genomic DNA with primers A and B as *Eco*RI-*Kpn*I fragment	This study
pME6032::$natT_{I142M\ S170T}$	pME6032 with $natT_{I142M\ S170T}$ under the control of *lac* promoter	*natT* (PA1030) was amplified from CI_1178 genomic DNA with primers A and B as *Eco*RI-*Kpn*I fragment	This study
pME6032::$natT_{T100A\ S170T}$	pME6032 with $natT_{T100A\ S170T}$ under the control of *lac* promoter	*natT* (PA1030) was amplified from CI_1138 genomic DNA with primers A and B as *Eco*RI-*Kpn*I fragment	This study
pME6032::$natT_{I14V\ V117A\ P165S}$	pME6032 with $natT_{I14V\ V117A\ P165S}$ under the control of *lac* promoter	*natT* (PA1030) was amplified from CI_1124 genomic DNA with primers A and B as *Eco*RI-*Kpn*I fragment	This study
pME6032::$natT_{I14V\ V117A\ S170T}$	pME6032 with $natT_{I14V\ V117A\ S170T}$ under the control of *lac* promoter	*natT* (PA1030) was amplified from CI_1128 genomic DNA with primers A and B as *Eco*RI-*Kpn*I fragment	This study
pME6032::$natT_{I14V\ V117A\ S232A}$	pME6032 with $natT_{I14V\ V117A\ S232A}$ under the control of *lac* promoter	*natT* (PA1030) was amplified from PA14 genomic DNA with primers A and B as *Eco*RI-*Kpn*I fragment	This study
pME6032::$natT_{I14V\ S105C\ V117A\ P165S\ S232A}$	pME6032 with $natT_{I14V\ S105C\ V117A\ P165S\ S232A}$ under the control of *lac* promoter	*natT* (PA1030) was amplified from CI_1179 genomic DNA with primers A and B as *Eco*RI-*Kpn*I fragment	This study

pME6032::*natT*$_{I14V\ S105C\ V117A\ E167K\ S170T}$	pME6032 with *natT*$_{I14V\ S105C\ V117A\ E167K\ S170T}$ under the control of *lac* promoter	*natT* (PA1030) was amplified from CI_1160 genomic DNA with primers A and B as *EcoR*I-*Kpn*I fragment	This study
pME6032::*natT*$_{wt}$-*FLAG*	pME6032 with C-terminally 3xFLAG-tag *natT* under the control of *lac* promoter	*natT* (PA1030) was amplified from PAO1 genomic DNA with primers A and F as *EcoR*I-*Kpn*I fragment	This study
pME6032::*natT*$_{E29D}$-*FLAG*	pME6032 with C-terminally 3xFLAG-tag *natT*$_{E29D}$ under the control of *lac* promoter	*natT* (PA1030) was amplified from PAO1 genomic DNA with primers A and F as *EcoR*I-*Kpn*I fragment	This study
pGn6032	GmR, pME6032 based vector, Tet cassette replaced with *aacCI* gentamycin resistance cassette	-	6
pGn6032::*natT*$_{wt}$	pGn6032 with *natT*$_{wt}$ under the control of *lac* promoter	*natT* (PA1030) was amplified from PAO1 genomic DNA with primers A and B as *EcoR*I-*Kpn*I fragment	This study
pGn6032::*natT*$_{E29D}$	pGn6032 with *natT*$_{E29D}$ under the control of *lac* promoter	*natT* (PA1030) was amplified from *natT*$_{E29D}$ genomic DNA with primers A and B as *EcoR*I-*Kpn*I fragment	This study
pQF	Tet^{R}, pQF based vector		7
pQF::*natR*	pQF with *natR* under the control of cumate-inducible promoter	*natR* (PA1029) was amplified from PAO1 genomic DNA with primers G and H as *Hind*III-*Kpn*I fragment	This study
pRSF-Duet-1_His-NatT-NatR	pRSF-Duet-1 (Novagen) with N-terminally His-tag NatT and NatR under the control of *lac* promoter	*natT* (PA1030) was amplified with primers I and J as *BamH*I-*EcoR*I fragment and *natR* (PA1029) was amplified with primers K and E as *EcoRV*-*Kpn*I fragment from PAO1 genomic DNA	This study
pRSF-Duet-1_His-NatT$_{E29D}$-NatR	pRSF-Duet-1 (Novagen) with N-terminally His-tag NatT$_{E29D}$ and NatR under the control of *lac* promoter	*natT* (PA1030) was amplified with primers I and J as *BamH*I-*EcoR*I fragment and *natR* (PA1029) was amplified with primers K and E as *EcoRV*-*Kpn*I fragment from PAO1 genomic DNA	This study
**Deletion constructs**			
pEX18-Tc	*oriT*+, *sacB*+, gene replacement vector, Tc^{R}	-	8
pEX18-Tc-*natT*$_{E29D}$	pEX18-Tc carrying the G to T in the *natT* allele as *Hind*III-*Xba*I fragment	-	2
pEX18-Tc-*natT*$_{E29D\ R82A}$	pEX18-Tc carrying the G to T and CGG to GCC in the *natT* allele as *Hind*III-*Xba*I fragment	Point mutation inserted into pEX18-Tc-*natT*$_{E29D}$ with primers L and M	This study
pEX18-Tc-Δ*natT*	pEX18-Tc carrying *natT* (PA1030) deletion cassette as *Hind*III-*Kpn*I fragment, Tet^{R}	Deletion cassette generated by ligation of upstream fragment (primers N and O, *Hind*III-*Xba*I) and downstream fragment (primers P and Q, *Xba*I-*Kpn*I) amplified from PAO1 gDNA	This study
pEX18-Tc-Δ*natR*	pEX18-Tc carrying *natR* (PA1029, full length corrected gene) deletion cassette as *Hind*III-*Kpn*I fragment, Tet^{R}	Deletion cassette generated by ligation of upstream fragment (primers T and U, *Hind*III-*Xba*I) and downstream fragment (primers R and S, *Xba*I-*Kpn*I) amplified from PAO1 gDNA	This study
pEX18-Tc-Δ*natRT*	pEX18-Tc carrying *natR* (PA1029, full length corrected gene) and natT (PA1030) deletion cassette as *Hind*III-*Kpn*I fragment, Tet^{R}	Deletion cassette generated by ligation of upstream fragment (primers T and U, *Hind*III-*Xba*I) and downstream fragment (primers P and Q, *Xba*I-*Kpn*I) amplified from PAO1 gDNA	This study
pEX18-Tc-Δ*relA*	pEX18-Tc carrying *relA* (PA0934) deletion cassette as *Hind*III-*Kpn*I fragment, Tet^{R}	Deletion cassette generated by ligation of upstream fragment (primers V and W, *Hind*III-*Xba*I) and downstream fragment (primers X and Y, *Xba*I-*Kpn*I) amplified from PAO1 gDNA	This study

pEX18-Tc-Δ*spoT*	pEX18-Tc carrying *spoT* (PA5338) deletion cassette as *Hin*dIII-*Kpn*I fragment, Tet^R	Deletion cassette generated by ligation of upstream fragment (primers Z and AB, *Hin*dIII-*Xba*I) and downstream fragment (primers AC and AD, *Xba*I-*Kpn*I) amplified from PAO1 gDNA	This study
pEX18-Tc-Δ*nrtR*	pEX18-Tc carrying *nrtR* (PA4916) deletion cassette as *Hin*dIII-*EcoR*I fragment, Tet^R	Deletion cassette generated by ligation of upstream fragment (primers AF and AG, *Hin*dIII-*Xba*I) and downstream fragment (primers AH and AI, *Xba*I-*EcoR*I) amplified from PAO1 gDNA	This study
pEX18-Tc-Δ*nadD2*	pEX18-Tc carrying *nadD2* (PA4917) deletion cassette as *Hin*dIII-*Kpn*I fragment, Tet^R	Deletion cassette generated by ligation of upstream fragment (primers AJ and AK, *Hin*dIII-*Xba*I) and downstream fragment (primers AL and AM, *Xba*I-*Kpn*I) amplified from PAO1 gDNA	This study
pEX18-Tc-*pcnB1::gfp*	pEX18-Tc carrying construct for chromosomal insertion the *gfp* gene with its own Shine-Dalgarno downstream *pcnB1* (PA4919) gene as *BamH*I-*Kpn*I fragment, Tet^R	Fragment generated by ligation of *gfp* containing fragment (from miniCTX::*pX2-gfp* as *BamH*I-*Kpn*I) and upstream fragment (primers AN and AO, *Hind*III-*BamH*I) and downstream fragment (primers AP and AQ, *SphH*I-*Kpn*I) amplified from PAO1 gDNA	This study
pEX18-Tc-*natRT::gfp*	pEX18-Tc carrying construct for chromosomal insertion the *gfp* gene with its own Shine-Dalgarno downstream *natT* gene as *BamH*I-*Kpn*I fragment, Tet^R	Fragment generated by ligation of *gfp* containing fragment (from miniCTX::*pX2-gfp* as *BamH*I-*Kpn*I) and upstream fragment (primers AR and AS, *Hind*III-*BamH*I) and downstream fragment (primers N and AT, *Xho*I-*Kpn*I) amplified from PAO1 gDNA	This study
pEX18(Gm)::del_fliC	pEX18-Gm carrying construct for *fliC* deletion with HindIII and KpnI sites	Amplification of 700 bp upstream and downstream of the target gene by SOE PCR	This study
pEX18-Tc-Δ*natT::gfp*	pEX18-Tc carrying *natT* (PA1030) deletion cassette as *Hin*dIII-*Kpn*I fragment for deletion of *natT* in $natRT_{E29D}$*::gfp* strain, Tet^R	Deletion cassette generated by ligation of upstream fragment (primers AJ and AK, *Hin*dIII-*Xba*I) and downstream fragment (primers AV and AU, *Xba*I-*Kpn*I) amplified from *natRT::gfp* gDNA	This study
pEX18-Tc- *natT-FLAG*	pEX18-Tc carrying construct for chromosomal insertion of 3xFLAG-tag at the C- terminus of NatT as *BamH*I-*Kpn*I fragment, Tet^R	Fragment generated by ligation of upstream fragment (primers AR and AW, *Hin*dIII-*Xba*I) and downstream fragment (primers P and Q, *Xba*I-*Kpn*I) amplified from *natRT::gfp* gDNA	This study
pME3087::pilA-KO	pME3087 carrying pilA (PA4525) deletion cassette as BamHI-EcoRI fragment (TetR)	SOE PCR with primers 5710 and 5711 for the upstream fragment, 5712 and 5713 for the downstream fragment	9

Appendix Table S3. Oligos used in this study

Primers	Description	Sequence
A	NatT_over_F	cca**GAATTC**GTGAGCGAGATCTGGCGACAGTGCAA
B	NatT_over_R	cc**GGTACC**TCACGCCGGATGCGGCAACTTTCCGT
C	$NatT_{ATG}$_over_F	cca**GAATTC**ATGAGCGAGATCTGGCGACAGTGCAA
D	NatR_over_F	cca**GAATTC**ATGAGCGCTCTCATCAAGGAACGTCC
E	NatR_over_R	cc**GGTACC**TCAGACCTTGCCGCGGATCGCATCGA
F	NatT_FLAG_R	cc**GGTACCTCACTTGTCATCGTCATCCTTGTAATCGA TATCATGATCTTTATAATCACCGTCATGGTCTTTGTAGTC**C GCCGGATGCGGCAACTTTCCGTCCA
G	pQF_natR_F	gat**AAGCTT**ATGAGCGCTCTCATCAAGGAACGTC
H	pQF_natR_R	gcg**GGATCC**TCAGACCTTGCCGCGGATCGCATC
I	RSF_His30F	gcg**GGATCC**GAGCGAGATCTGGCGACAGTGCAAGGG
J	RSF_His30R	cca**GAATTC**TCACGCCGGATGCGGCAACTTTCCGT
K	RSF_29F	cga**GATATC**GAGCGCTCTCATCAAGGAACGTCCCAG
L	NatT_R82A_F	CTTGCGCTGGGGGTCG**gcc**TTTGGCCGTCGCC
M	NatT_R82A_F	AAA**ggc**CGACCCCCAGCGCAAGGGCGGATAACG
N	NatT_KO_UPF	gat**AAGCTT**CTCTCTATCACGCCGGTCAATGCTTG
O	NatT_KO_UPR	cg**TCTAGA**CTTCCGCCCCTCGCTTCCTGTGGTGT
P	NatRT_KO_DnF	cg**TCTAGA**GTGGAGGGGGCTCGGTTGCCAGCGAT
Q	NatRT_KO_DnR	gc**GGTACC**GATGGAGTGGGGCTATGGGACAGATT
R	NatR_KO_DnF	cg**TCTAGA**TACCCGGCGCGCCTGTACGGGGGCCT
S	NatR_KO_DnR	gc**GGTACC**CGCTGCGCATGGCACTTCCCAGTTCC
T	NatRT_KO_UPF	gat**AAGCTT**AGGGTTCCTGGGATACCAGGACGGTG
U	NatRT_KO_UPR	cg**TCTAGA**GACGACCTCCTCATGTGTGTTTACA
V	relA_ko_UPF	gat**AAGCTT**ACTGGCAACTGACTCTGGCATA
W	relA_ko_UPR	cg**TCTAGA**CATCTTGCCTACCCTTTACCAC
X	relA_ko_DnF	cg**TCTAGA**TGAGGCGAGGCGGAAACAGGCC
Y	relA_ko_DnR	gc**GGTACC**TTCGTCCAGCCCCTCGCGAACC
Z	spoT_KO_UPF	gat**AAGCTT**GCCCAGCAGGTGCGCCGGCTGA
AB	spoT_KO_UPR	cg**TCTAGA**CAAGGGTTCACCCCCTGCCCGT
AC	spoT_KO_DnF	cg**TCTAGA**TGACCCGCTTTTTCCTGTGTCA
AD	spoT_KO_DnR	gc**GGTACC**CAGTTCGAAGCCGTTGCTGAAG
AF	nrtR_KO_UPF	gat**AAGCTT**CATGAATCGGTGATCCGCCGGGCGC
AG	nrtR_KO_UPR	cg**TCTAGA**CATCGTCACTCCTCTCTTCAGACCG
AH	nrtR_KO_DnF	cg**TCTAGA**TGACAAACGGGTGCGGCAGGGCCGG
AI	nrtR_KO_DnR	cca**GAATTC**TGCCGCGATAGAAGCCGCACGGGCG
AJ	PA4917_KO_UPF	gat**AAGCTT**CACATGGCTCACGCCATTCTGCCTC
AK	PA4917_KO_UPR	cg**TCTAGA**AGAAAATACCTTCCACTGCGAAATTTTG
AL	PA4917_KO_DnF	cg**TCTAGA**AGAGAGGAGTGACGATGAGTTCAGC
AM	PA4917_KO_DnR	gc**GGTACC**CTGGACTTCCTGGCCGATGGCGCAC
AN	Pa4919GFP_UPF	gat**AAGCTT**CCGCACCCAGGAGGAAGTGGTGCAT
AO	Pa4919GFP_UPR	gcg**GGATCC**TTAGGCCGGCACGTTGAAGACGTGG
AP	Pa4919GFP_DnF	cc**GCATGC**TCCTTTCGCACCTAACGGGAGCCCG

AQ	Pa4919GFP_DnR	cc**GGTACC**ATGCTTGGCCAGGCCGCTGAGCGGA
AR	10458_Pa1030GFP_UPF	gat**AAGCTT**AACAGGCGTTGCTCGAGGAACTGCTC
AS	10459_Pa1030GFP_UPR	gcg**GGATCC**TCACGCCGGATGCGGCAACTTTCCGT
AT	10602_Pa1030GFP_DnF	cca**CTCGAG**GTGGAGGGGGCTCGGTTGCCAGCGAT
AU	18532_30KO_DnF	cg**TCTAGA**GGATCCTCTAGATTTAAGAAGGAGATA
AV	18533_30KO_DnR	gc**GGTACC**AGGGCAGATTGTGTGGACAGGTAATG
AW	PA1030_C-FLAG-R	cg**TCTAGA**TCACTTGTCATCGTCATCCTTGT

Appendix Table S4. Crystallographic data collection and refinement statistics

	NatRT wt (PDB ID: 8QNL)	NatRT_E29D (PDB ID: 8QNQ)
**Resolution range**	53.4 - 2.2 (2.3 - 2.2)	53.7 - 2.3 (2.4 - 2.3)
**Space group**	P 1 21 1	P 1 21 1
**Unit cell**	55.1 106.9 93.49 90 104.2 90	55.4 107.7 93.5 90 104.0 90
**Total reflections**	330825	269486
**Unique reflections**	47668 (4791)	39154 (4174)
**Multiplicity**	6.9	6.9
**Completeness (%)**	98.88 (99.50)	92.23 (98.93)
**Mean I/sigma(I)**	13.0	11.8
**Wilson B-factor**	43.19	43.74
**R-merge**	0.09	0.225
**R-pim**	0.04	0.094
**CC1/2**	0.99	0.97
**Reflections used in refinement**	47663 (4791)	39148 (4174)
**Reflections used for R-free**	2322 (254)	1971 (215)
**R-work**	0.20 (0.27)	0.23 (0.31)
**R-free**	0.25 (0.34)	0.26 (0.35)
**Number of non-hydrogen atoms**	7857	7812
**macromolecules**	7708	7730
**ligands**	20	20
**solvent**	129	62
**Protein residues**	980	985

**RMS(bonds)**	0.003	0.004
**RMS(angles)**	0.59	0.69
**Ramachandran favored (%)**	98.04	96.51
**Ramachandran allowed (%)**	1.76	2.88
**Ramachandran outliers (%)**	0.21	0.62
**Rotamer outliers (%)**	2.00	0.12
**Clashscore**	4.18	7.59
**Average B-factor**	52.07	51.72
**macromolecules**	52.23	51.85
**ligands**	42.38	38.85
**solvent**	43.91	39.79

Statistics for the highest-resolution shell are shown in parentheses.

Appendix Figure S1. Phylogenetic tree of bacterial RES domain proteins

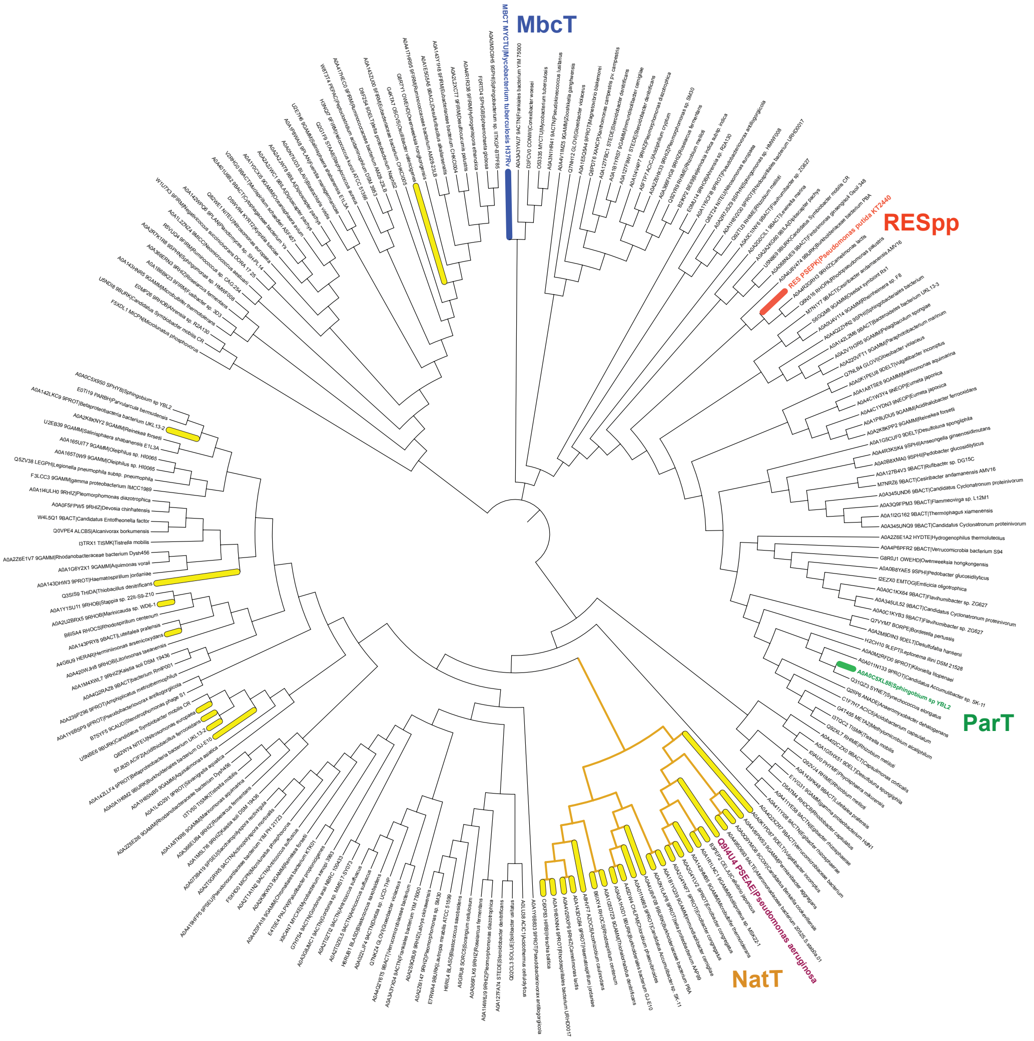

Phylogenetic tree of bacterial RES domain proteins. NatT, ParT, RESpp and MbcT are shown. Branches of RES domains containing a Flap region are highlighted in yellow.

References

1. Holloway, B. W. Genetic Recombination in *Pseudomonas aeruginosa*. *Microbiology* **13**, 572–581 (1955).

2. Santi, I., Manfredi, P., Maffei, E., Egli, A. & Jenal, U. Evolution of Antibiotic Tolerance Shapes Resistance Development in Chronic *Pseudomonas aeruginosa* Infections. *mBio* **12**, e03482-20 (2021).

3. Manner, Christina et al. "A genetic switch controls Pseudomonas aeruginosa surface colonization." Nature microbiology vol. 8,8 (2023): 1520-1533. doi:10.1038/s41564-023-01403-0

4. Held, K., Ramage, E., Jacobs, M., Gallagher, L. & Manoil, C. Sequence-Verified Two-Allele Transposon Mutant Library for *Pseudomonas aeruginosa* PAO1. *J. Bacteriol.* **194**, 6387–6389 (2012).

5. Heeb, S. *et al.* Small, Stable Shuttle Vectors Based on the Minimal pVS1 Replicon for Use in Gram-Negative, Plant-Associated Bacteria. *Mol. Plant-Microbe Interact.* **13**, 232–237 (2000).

6. Malone, J. G. *et al.* The YfiBNR Signal Transduction Mechanism Reveals Novel Targets for the Evolution of Persistent *Pseudomonas aeruginosa* in Cystic Fibrosis Airways. *PLoS Pathog* **8**, e1002760 (2012).

7. Klotz, A., Kaczmarczyk, A. & Jenal, U. A Synthetic Cumate-Inducible Promoter for Graded and Homogenous Gene Expression in *Pseudomonas aeruginosa*. *Appl. Environ. Microbiol.* **89**, e00211-23 (2023).

8. Voisard, C. *et al.* Molecular Ecology of Rhizosphere Microorganisms. 67–89 (2010) https://doi.org/10.1002/9783527615810.ch6.

9. Laventie, Benoît-Joseph et al. A Surface-Induced Asymmetric Program Promotes Tissue Colonization by Pseudomonas aeruginosa. *Cell host & microbe* vol. 25,1 (2019): 140-152.e6. doi:10.1016/j.chom.2018.11.008
