# Supplementary material for: Toxin-mediated depletion of NAD and NADP drives persister formation in a human pathogen
Source: EMBO J. 2024 Sep 25;43(21):19. doi: 10.1038/s44318-024-00248-5 (PMC11535050; doi:10.1038/s44318-024-00248-5)
Supplement: Supplementary file 4 — Movie EV1 [file 44318_2024_248_MOESM4_ESM.zip › Movie EV1.docx]

**Movies EV1-2.** Time-lapse microscopy of the *natT_E29D_* mutant strain harboring the *pncB1-gfp* reporter.
