# Supplementary material for: Toxin-mediated depletion of NAD and NADP drives persister formation in a human pathogen
Source: EMBO J. 2024 Sep 25;43(21):19. doi: 10.1038/s44318-024-00248-5 (PMC11535050; doi:10.1038/s44318-024-00248-5)
Supplement: Supplementary file 13 — Expanded View Figures [file 44318_2024_248_MOESM13_ESM.pdf]

Expanded View Figures

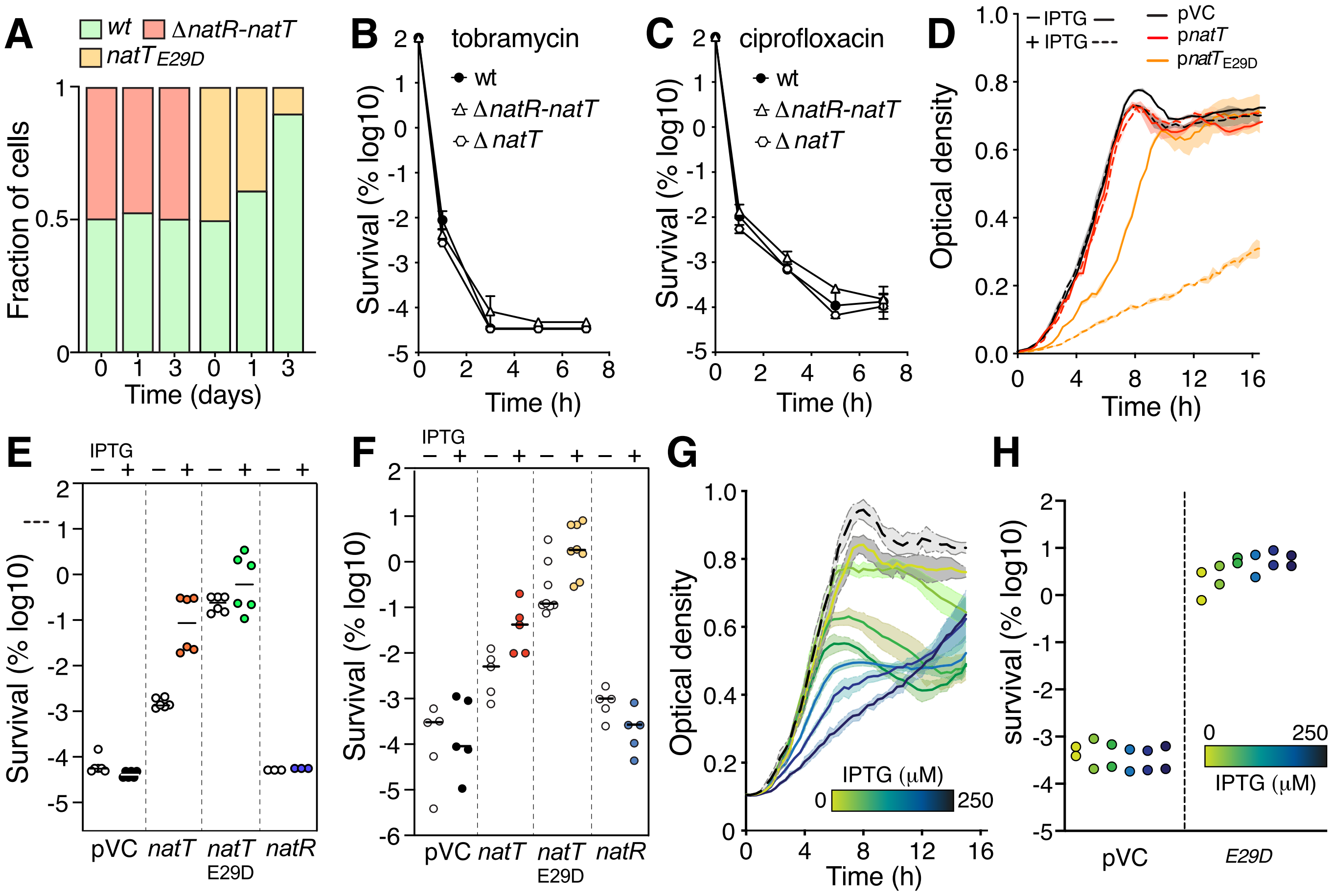

Figure EV1. The NatT toxin confers drug tolerance to *P. aeruginosa*.

(**A**) Reduced fitness of a *P. aeruginosa* $natT_{E29D}$ mutant. *P. aeruginosa* strains expressing different fluorophores were mixed 1:1 and subjected to consecutive cycles of growth and re-dilution. Subpopulations were analyzed by flow cytometry at indicated time intervals. (**B**, **C**) Survival of Δ*natT* and Δ*natR*-Δ*natT* mutants during treatment with tobramycin (8 μg/ml) (**B**) or ciprofloxacin (2.5 μg/ml) (**C**) (technical replicates, average ± SEM, $n = 3$). (**D**) Expression of *natT* impairs *P. aeruginosa* growth. Cultures of a Δ*natT* mutant containing plasmids with IPTG-inducible *natT* alleles were grown in LB medium with or without IPTG (average ± SEM, $n > 3$) (pVC = control plasmid). (**E**) Expression of *natT* increases tolerance to tobramycin. Cultures of *P. aeruginosa* containing plasmids with IPTG-inducible *natT* or *natR* alleles were grown in LB medium without or with IPTG. Fractions of surviving cells were determined after three hours of treatment with tobramycin (16 μg/ml). Median values are indicted ($n \geq 3$) (pVC = control plasmid). (**F**) Expression of *natT* increases tolerance to ciprofloxacin. Cultures of *P. aeruginosa* containing plasmids with IPTG-inducible *natT* or *natR* alleles were grown in LB medium without (empty boxes) or with 250 μM IPTG (filled boxes). Fractions of surviving cells were determined after three hours of treatment with ciprofloxacin (2.5 μg/ml) ($n \geq 5$; lines mark medians) (pVC = control plasmid). (**G**) Expression of $natT_{E29D}$ gradually limits *P. aeruginosa* growth. Cultures of a Δ*natT* mutant containing a control plasmid (stippled line) or a plasmid expressing $natT_{E29D}$ from an inducible promoter, were grown in LB with increasing concentrations of IPTG as indicated (average ± SEM, $n > 3$). (**H**) Expression of $natT_{E29D}$ increases *P. aeruginosa* tolerance. *P. aeruginosa* cultures containing a plasmid with an IPTG-inducible $natT_{E29D}$ were grown as in (**G**) and survival was determined after three hours of treatment with tobramycin (16 μg/ml) (average ± SEM).

© The Author(s)

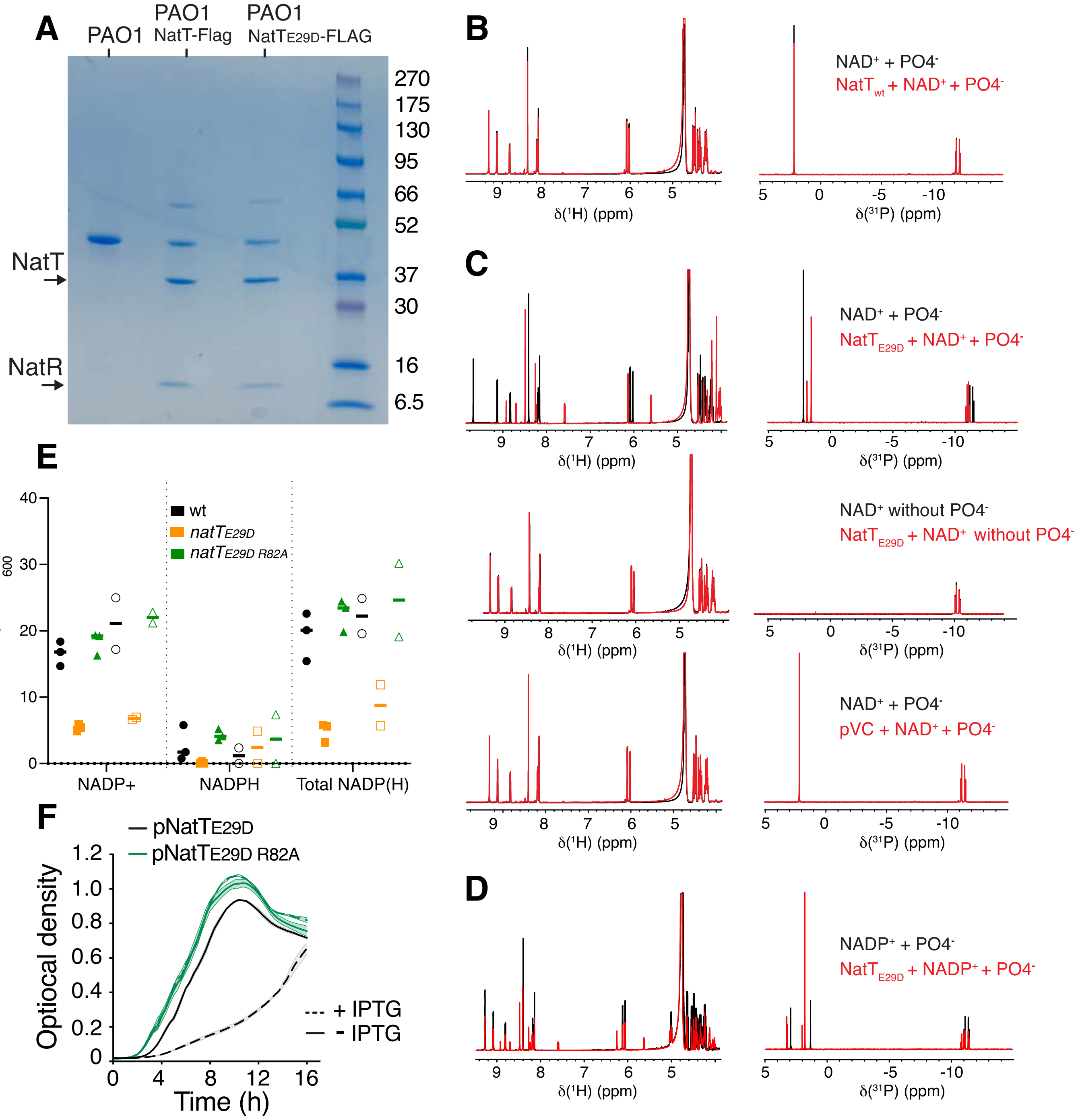

Figure EV2. NatT is a NAD-dependent phosphorylase.

(**A**) Purification of NatR–NatT complex from *P. aeruginosa*. NatR and NatT proteins were copurified from a *P. aeruginosa ΔnatT* mutants harboring plasmids containing FLAG-tagged *natT* alleles by affinity chromatography using anti-FLAG beads. (**B**) Degradation of NAD^+ by $NatT_{E29D}$ is phosphate-dependent. Purified NatR-$NatT_{E29D}$ complex was incubated with NAD^+ with and without phosphate, and the reaction was analyzed by 1D ^1H and ^{31}P NMR spectra. (**C**) Purified NatR–NatT wild-type does not degrade NAD^+. (**D**) Purified NatR-$NatT_{E29D}$ degrades $NADP^+$ in a phosphate-dependent reaction. (**E**) NatT depletes cellular NADP pools of *P. aeruginosa*. $NADP^+$ and NADPH concentrations were determined in cultures of *P. aeruginosa* wild-type (wt) and in strains harboring plasmids expressing $natT_{E29D}$ or $natT_{E29D\ R82A}$ growing exponentially (filled bars) or from stationary phase (open bars) (average ± SEM, $n = 2$). (**F**) The active site residue R82 is required for NatT-mediated toxicity. *P. aeruginosa* with plasmids containing IPTG-inducible copies of $natT_{E29D}$ (black line) or $natT_{E29D\ R82A}$ (green line) were grown in LB with (dotted lines) or without IPTG (solid lines) (average ± SEM, $n = 3$).

 © The Author(s)

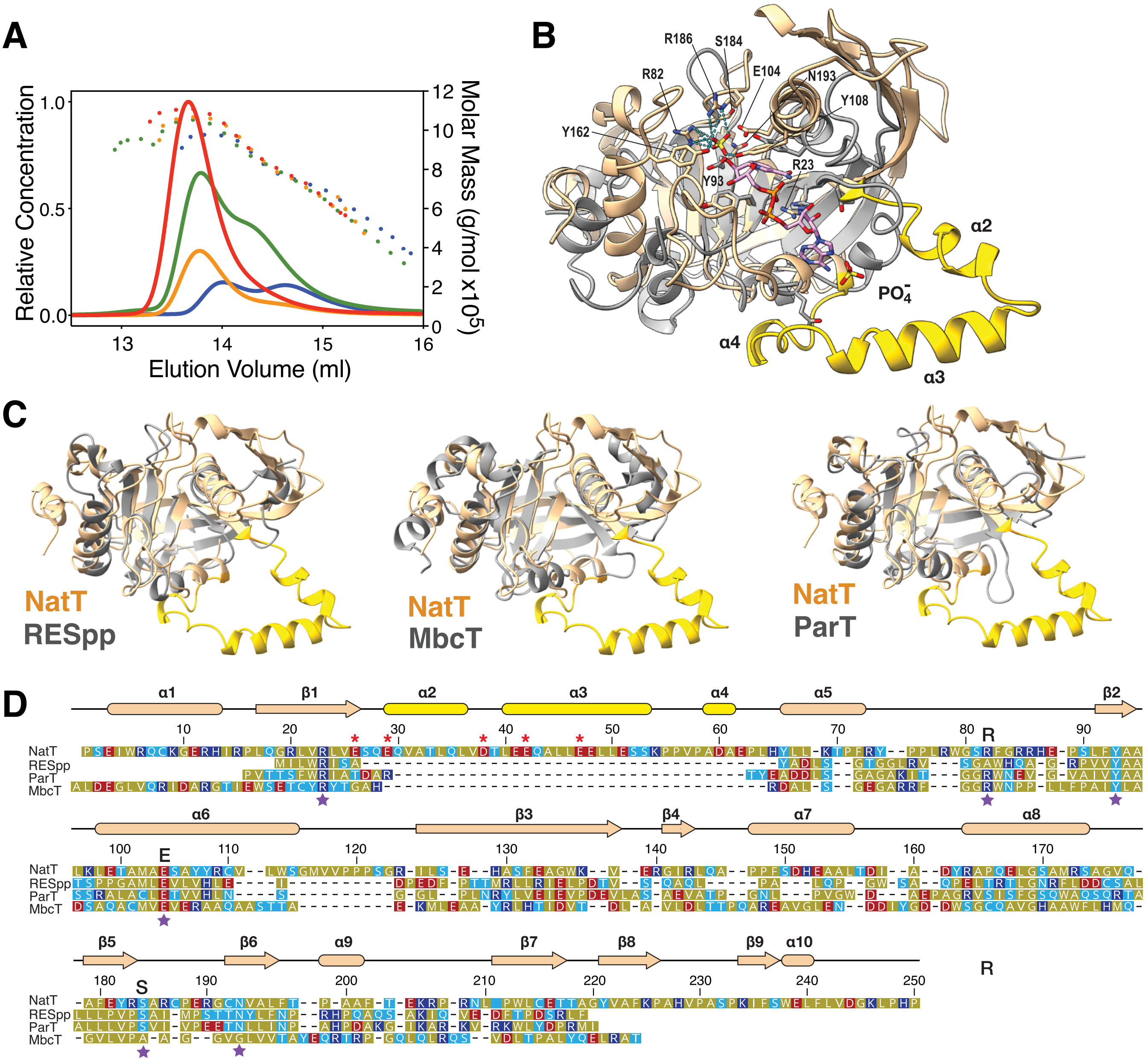

Figure EV3. Interaction of NatR and NatT mediates toxin activation.

(**A**) SEC-MALS analysis of NatR-NatT and NatR-$NatT_{E29D}$ complexes reveal different oligomeric structures. Experiments were performed with 7 μM (red) and 2 μM (orange) NatR-NatT and with 7 μM (green) and 2 μM (blue) NatR-$NatT_{E29D}$. (**B**) Structural homology between NatT and diphtheria toxin indicates analogous NAD^+ binding modes. NatT is shown in light brown and yellow (Flap), diphtheria toxin (1tox) is in gray and its active site NAD^+ is in pink. (**C**) Superposition of *P. aeruginosa* NatT (light brown) with Flap (yellow) and RES domain proteins RESpp (Skjerning et al, 2019), MbcT (Freire et al, 2019) and ParT (Piscotta et al, 2019) (gray). (**D**) Structure-guided sequence alignment of NatT and RES domain proteins shown in (**B**). Predicted active site residues are marked with purple stars. Charged residues of the Flap involved in interaction with NatR′ are marked with red asterisks.

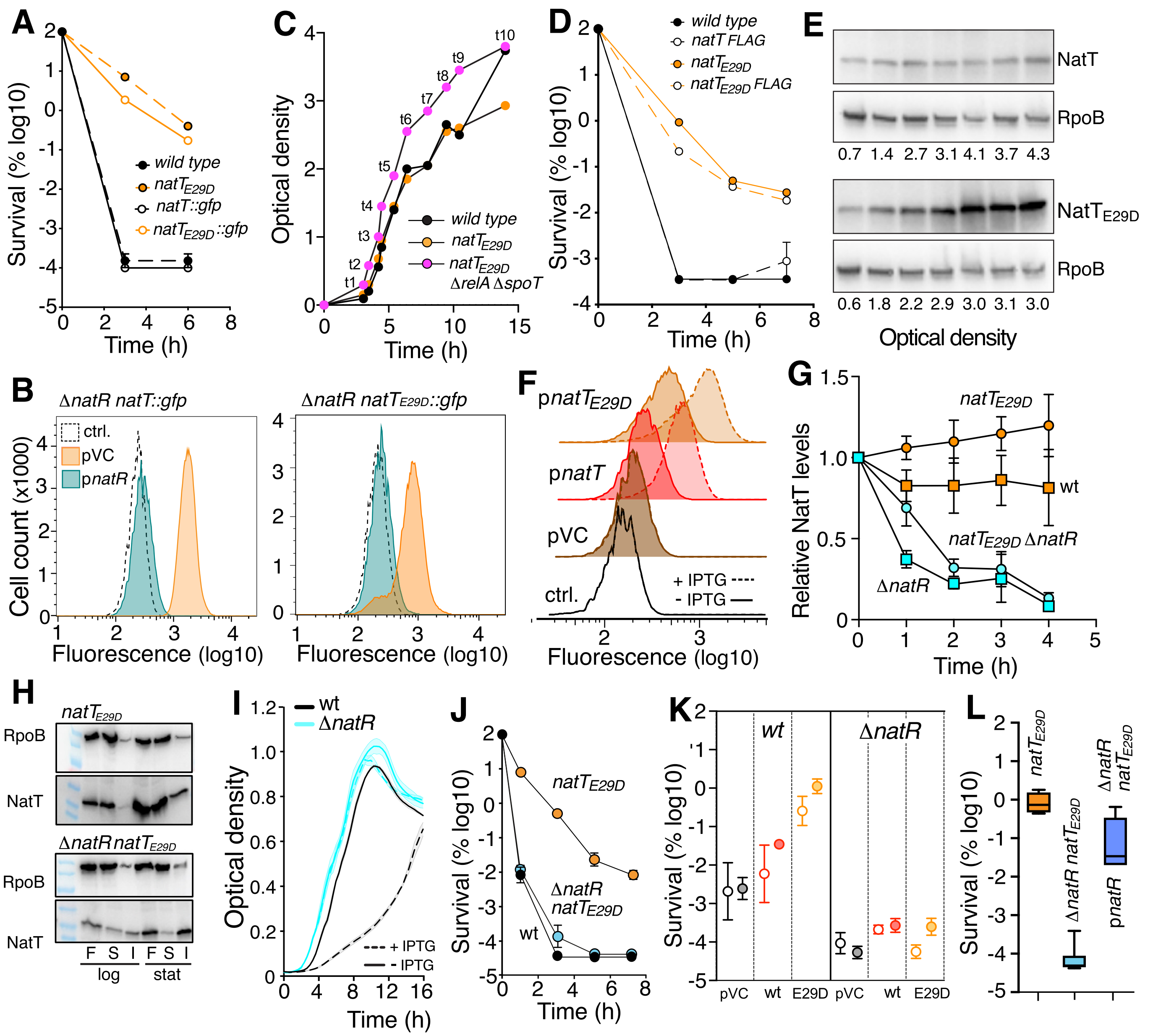
A
Survival (% log10)
Time (h)
wild type
natTE29D
natT::gfp
natTE29D::gfp
C
Optical density
Time (h)
wild type
natTE29D
natTE29D ΔrelA ΔspoT
D
Survival (% log10)
Time (h)
wild type
natT FLAG
natTE29D
natTE29D FLAG
E
NatT
RpoB
0.7 1.4 2.7 3.1 4.1 3.7 4.3
NatTE29D
RpoB
0.6 1.8 2.2 2.9 3.0 3.1 3.0
Optical density
B
ΔnatR natT::gfp
ΔnatR natTE29D::gfp
ctrl.
pVC
pnatR
Cell count (x1000)
Fluorescence (log10)
F
pnatTE29D
pnatT
pVC
ctrl.
+ IPTG
- IPTG
Fluorescence (log10)
G
Relative NatT levels
natTE29D
wt
natTE29D ΔnatR
ΔnatR
Time (h)
H
natTE29D
RpoB
NatT
ΔnatR natTE29D
RpoB
NatT
F S I F S I
log stat
I
wt
ΔnatR
Optical density
+ IPTG
- IPTG
Time (h)
J
Survival (% log10)
natTE29D
ΔnatR natTE29D
wt
Time (h)
K
Survival (% log10)
wt
ΔnatR
pVC wt E29D pVC wt E29D
L
Survival (% log10)
natTE29D
ΔnatR natTE29D
ΔnatR natTE29D
pnatR

 © The Author(s)

Figure EV4. NatR is an anti- and a co-toxin of NatT.

(**A**) Introduction of a chromosomal *natT*::*gfp* reporter does not affect *P. aeruginosa* drug tolerance. Survival of *P. aeruginosa* was scored during exposure to tobramycin (16 μg/ml) (technical replicates, average ± SEM, $n > 3$). (**B**) NatR is a repressor of *natT* transcription. Transcription of *natT* was determined by flow cytometry in Δ*natR* mutants with a *gfp* reporter downstream of *natT*. Strains contained a control plasmid (orange) or a plasmid expressing *natR* (green). Control strains lacking the *gfp* reporter are indicated by a dotted black line. (**C**) Growth of *P. aeruginosa* wild-type, $natT_{E29D}$ and $natT_{E29D}$ Δ*relA* Δ*spoT* in LB. Samples from different time points (t1-t10) were used for flow citometry analysis in Fig. 4E ($n = 1$). (**D**) Drug tolerance is not affected by an engineered chromosomal *natT-FLAG* allele (technical replicates, average ± SEM, $n = 3$). (**E**) NatT protein levels increase in *P. aeruginosa* $natT_{E29D}$ upon entry into stationary phase. Levels of NatT and $NatT_{E29D}$ were determined during growth (see: **C**) by immunoblot analysis using anti-FLAG antibodies and anti-RpoB antibodies as control. (**F**) Ectopic expression of *natT* leads to derepression of *natR–natT* transcription. Transcription of *natR–natT* was determined in *P. aeruginosa* harboring a chromosomal *natT*::*gfp* reporter and plasmids with IPTG-inducible *natT* alleles as indicated (pVC = control plasmid). (**G**) NatT is degraded in strains lacking NatR. Concentrations of NatT were determined by immunoblot analysis after treating *P. aeruginosa* cultures with chloramphenicol and plotted as relative values of the initial concentration (0 h) (technical replicates, average ± SEM, $n \geq 3$). (**H**) NatT is insoluble in strains lacking NatR. NatT protein was quantified by immunoblot analysis of fractions harvested from different *P. aeruginosa* strains and in different growth phases as indicated. F: full lysate; S: soluble fraction; I: insoluble fraction. (**I**) NatT-mediated toxicity is abolished in a Δ*natR* mutant. Growth of *P. aeruginosa* wild-type and Δ*natR* mutant carrying a plasmid with an IPTG-inducible $natT_{E29D}$ allele was recorded in LB medium with or without IPTG as indicated (average ± SEM, $n = 3$). (**J**) NatR is required for NatT-mediated drug tolerance. Survival of *P. aeruginosa* wild-type and mutants indicated during exposure to tobramycin (average ± SEM, $n > 3$). (**K**) NatR is required for NatT-mediated drug tolerance. Survival of *P. aeruginosa* wild-type and Δ*natR* mutant carrying plasmids with IPTG-inducible copies of *natT* or $natT_{E29D}$ was determined after treatment with tobramycin (16 μg/ml). Cultures were grown with (filled circles) or without IPTG (empty circles) (average ± SEM, $n = 3$). (**L**) Ectopic expression of *natR* restores drug tolerance of a Δ$natR$–$natT_{E29D}$ mutant. Survival to tobramycin is shown for strains indicated. Plasmid p*natR* harbors an IPTG-inducible copy of *natR*. The box extends from the lower (25th percentile) to upper quartile (75th percentile) values, with a line at the median (50th percentile). Whiskers indicate the minimum and maximum values within 1.5 times the interquartile range ($n = 3$).

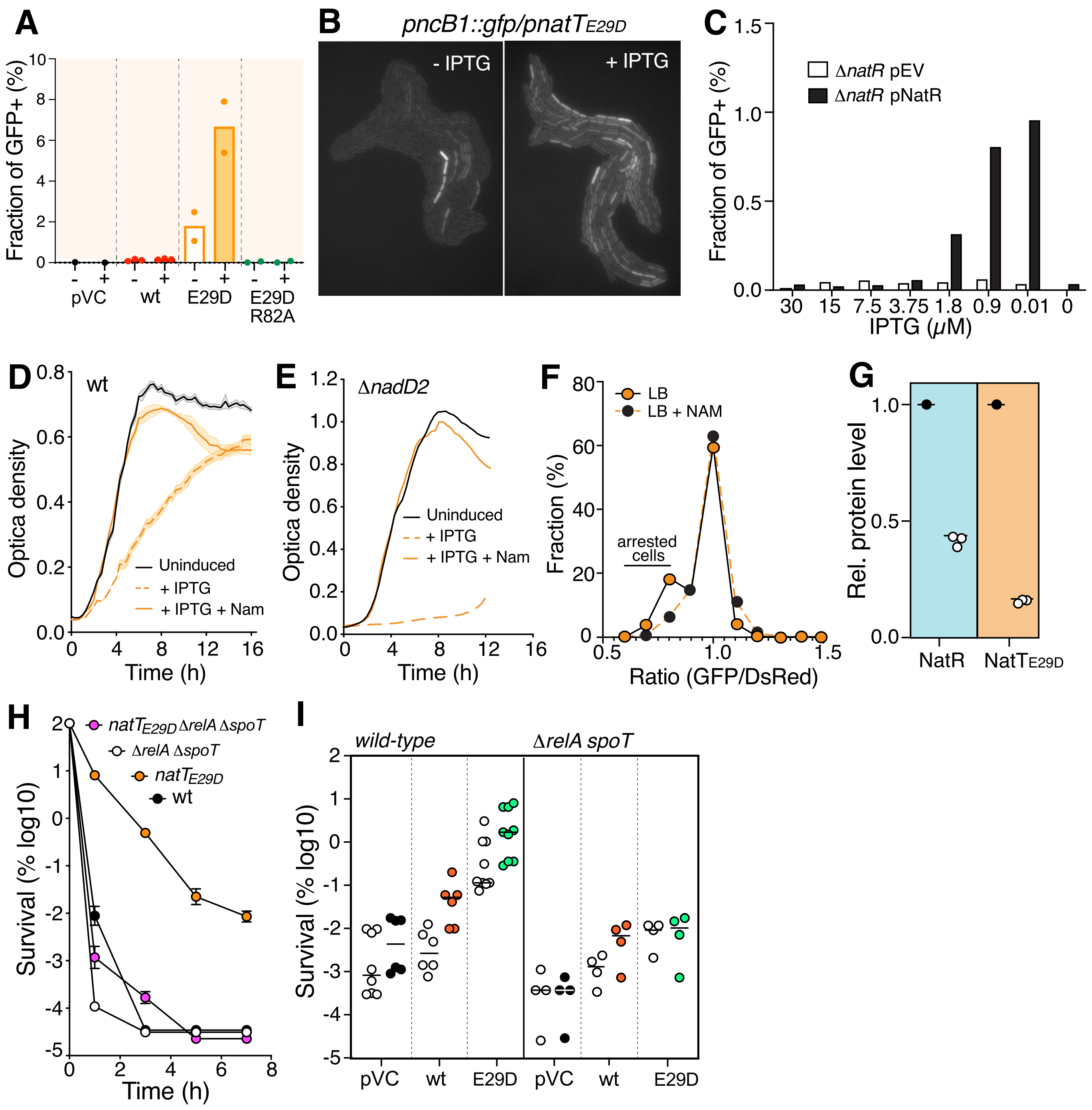
A
Fraction of GFP+ (%)
pVC
wt
E29D
E29D R82A
B
pncB1::gfp/pnatTE29D
- IPTG
+ IPTG
C
Fraction of GFP+ (%)
ΔnatR pEV
ΔnatR pNatR
30 15 7.5 3.75 1.8 0.9 0.01 0
IPTG (μM)
D
wt
Optica density
Uninduced
+ IPTG
+ IPTG + Nam
Time (h)
E
ΔnadD2
Optica density
Uninduced
+ IPTG
+ IPTG + Nam
Time (h)
F
LB
LB + NAM
Fraction (%)
arrested cells
Ratio (GFP/DsRed)
G
Rel. protein level
NatR
NatTE29D
H
natTE29D ΔrelA ΔspoT
ΔrelA ΔspoT
natTE29D
wt
Survival (% log10)
Time (h)
I
wild-type
ΔrelA spoT
Survival (% log10)
pVC
wt
E29D
pVC
wt
E29D

 © The Author(s)

Figure EV5. The NAD salvage pathway neutralizes NatT toxin activity and abolishes drug tolerance.

(**A**) Ectopic expression of *natT* mediates NAD^+ salvage pathway induction. Fractions of cells inducing salvage pathway genes were determined in *P. aeruginosa pncB1::gfp* reporter strains harboring plasmids expressing different *natT* alleles from an IPTG-inducible promoter. Cultures were grown with or without IPTG (average ± SEM, $n = 3$) (pVC control plasmid). (**B**) Ectopic expression $natT_{E29D}$ induces salvage pathway genes. Representative microscopy images of a *pncB1::gfp* reporter strain expressing $natT_{E29D}$ from a plasmid with or without IPTG. (**C**) Limiting *natR* expression induces salvage pathway genes. *P. aeruginosa* Δ*nrtR pncB1::gfp* carrying a plasmid with an IPTG-inducible *natR* was analyzed at different IPTG concentrations as indicated. Fractions of cells with derepressed salvage pathway were scored as a function of *natR* expression. (**D**, **E**) NAM neutralizes $NatT_{E29D}$-mediated growth defect in *P. aeruginosa* wild-type (**D**) or Δ*nadD2* salvage pathway mutant (**E**). Strains harboring a plasmid with an IPTG-inducible $natT_{E29D}$ allele were grown with (orange) or without IPTG (black) and with (solid lines) or without NAM (20 mM) (dotted lines). (**F**) NAM overrides NatT-mediated growth arrest. Cultures of *P. aeruginosa* wild-type and $natT_{E29D}$ mutant constitutively expressing TIMER were grown in LB with or without NAM for 3 h before analyzing populations by flow cytometry. Fractions of slow-growing or arrested cells were calculated using GFP/DsRed ratios of individual cells. (**G**) NAM limits NatR and NatT protein levels. A *P. aeruginosa* $natR_{E29D}$ mutant was grown in LB with (open circles) or without NAM (closed circles), followed by the analysis of relative levels of NatR and NatT by mass spectrometry (technical replicates, average ± SEM, $n = 3$). (**H**, **I**) (p)ppGpp is required for NatT-mediated drug tolerance. (**H**) Cultures of *P. aeruginosa* strains indicated were treated with tobramycin and survival was determined over time (technical replicates, average ± SEM, $n = 3$). (**I**) Survival of *P. aeruginosa* wild-type and Δ*relA* Δ*spoT* mutant carrying plasmids with IPTG-inducible *natT* (wt) or $natT_{E29D}$ (E29D) alleles was determined after three hours of treatment with tobramycin. Cultures were grown with (filled circles) or without IPTG (open circles). Solid lines mark median values.

© The Author(s)

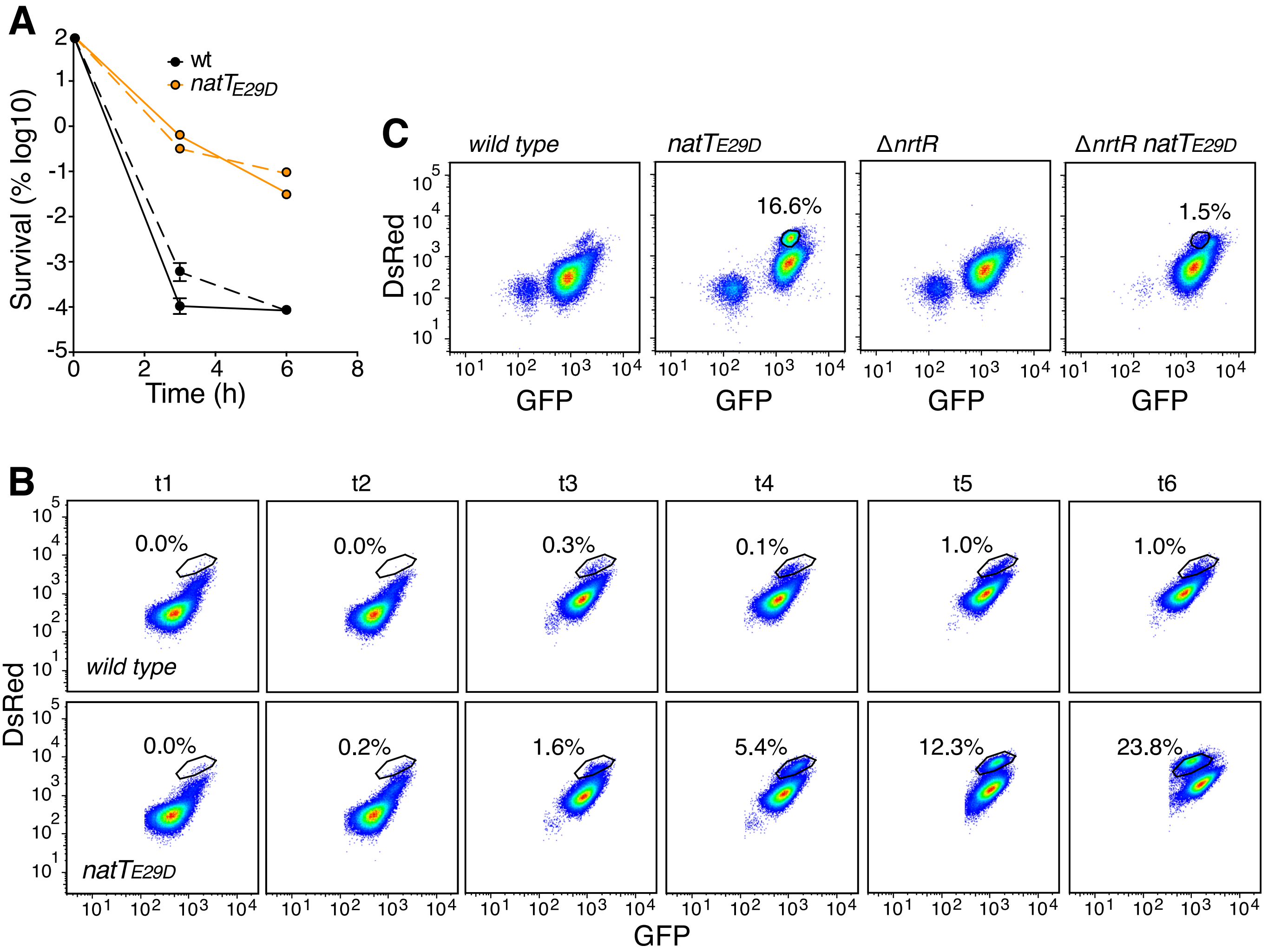

Figure EV6. NatT activation generates subpopulations with extended lag phase during outgrowth.

(**A**) Expression of TIMER from the chromosomal *attb* locus does not affect *P. aeruginosa* drug tolerance. Cell survival during tobramycin treatment was determined for isogenic strains with (dotted line) or without (solid line) TIMER (technical replicates, average ± SEM, $n = 3$). (**B**) The $natT_{E29D}$ allele induces growth arrest in a subpopulation of *P. aeruginosa* cells. Samples of *P. aeruginosa* wild-type and $natT_{E29D}$ mutant cultures expressing TIMER were harvested at different time points (see: Fig. 6A), diluted into fresh medium for three hours, and analyzed by flow cytometry. Fractions of slow-growing cells (black gate) are indicated. (**C**) Deletion of *nrtR* abolishes the population of slow-growing $natT_{E29D}$ mutant cells. Experiments were carried out as in (**B**) with cells being harvested at time point t5. Strains are indicated above the panels.

© The Author(s)
